# Perspectives on current recommendations for genetic testing in HCM

**DOI:** 10.21542/gcsp.2018.23

**Published:** 2018-08-12

**Authors:** Lorenzo Monserrat

**Affiliations:** Cardiology Department, Health In Code, A Coruña, Spain

## Introduction

Hypertrophic cardiomyopathy (HCM) is defined as a primary cardiac muscle disease characterized by the presence of myocardial hypertrophy in the absence of apparent causes for the observed degree of hypertrophy^1,2^. This definition includes both familial and sporadic (apparently non-familial) forms of the disease. HCM is usually considered as a genetically determined condition. Current genotyping technologies allow for the identification of the genetic causes of the disease in 50 to 70% of the patients who fulfill clinical diagnostic criteria. However, the etiology of 30 to 40% of the cases remains elusive^1,2^. This review is focused on the current role of genetic testing in HCM, and the potential benefits of the identification of the genetic etiology of the disease.

Genetic testing offers opportunities for a better understanding and management of HCM that can be summarized as follows:

1. Better diagnosis of index cases and relatives.
2. Better risk stratification.
3. More individualized therapies.
4. Better understanding of the disease.
5. Better genetic counseling^3,4^.

## Genetic testing in the diagnosis of index cases and relatives

HCM is a frequent condition in the general population, with an estimated prevalence of 1:500 adults. Even though the genetic cause of the disease is present from conception, the disease usually becomes apparent in the adolescence or later in life. An early diagnosis may be difficult, but it is very important, because HCM is one of the most frequent causes of unexpected sudden death in young individuals and athletes, and is also a relevant cause of unexpected sudden death, and cerebrovascular accidents at older ages^2^. The identification of a definitive genetic cause of the disease in the index patient provides a confirmation of the clinical diagnosis and may help in the differential diagnosis of the disease in the index. It also facilitates the cascade screening of the relatives, an early identification of those potentially affected, and the opportunity to stop unnecessary prolonged follow-up evaluations in non-carriers of the responsible genetic defects^1,5,6^.

### European Society of Cardiology guidelines for genetic testing

Current guidelines recommend genetic testing as a class I indication especially for the potential value of genetic testing in the familial screening, while the role of genetic testing in the diagnosis of the index case is less clearly acknowledged. The main recommendations and their implications are as follows^2^:

Recommendation of the ESC guideline on genetic testing in probands:

• “Genetic testing is recommended in patients fulfilling diagnostic criteria for HCM, when it enables cascade genetic screening of their relatives. Class I indication, B level of evidence”.
• “In the presence of symptoms and signs of disease suggestive of specific causes of HCM, genetic testing is recommended to confirm the diagnosis. Class I, level B.”

These two recommendations suggest that screening of relatives is the main indication of genetic testing in HCM cases, and could be interpreted as a lack of indication of genetic testing in patients without relatives at risk, unless a “specific cause of HCM” is suspected. In the second recommendation, the focus is on confirming the diagnosis of some rare conditions that cause a HCM phenotype and which may have a specific therapy or management, such as Fabry disease or familial amyloidosis. However, genetic diagnosis may have a more extended role.

• “It is recommended that genetic testing be performed in certified diagnostic laboratories with expertise in the interpretation of cardiomyopathy-related mutations. Class I, level C”.

This recommendation puts emphasis in the complexity of genetic testing performance and interpretation. Quality control is essential in sequencing and bioinformatics. Certifications usually cover these issues and decrease the risks of human and technical sequencing errors. Correct interpretation and of the results remains a major challenge and requires a considerable level of expertise and specialization.

• “Genetic testing in patients with a borderline diagnosis of HCM should be performed only after detailed assessment by specialist teams. Class IIa, level C”.

The yield of genetic testing and its predictive value depends on the pre-test probability of being affected by the condition. Genetic testing in patients with a borderline diagnosis provides a lower yield of positive findings and is also prone to a higher probability of false positive results. Expertise in the interpretation by the provider of the genetic test and by the requesting physicians is more critical than in cases with a clear diagnosis and higher pre-test probability. However, genetic testing should be performed after detailed assessment by specialist teams not only in borderline cases, but in all situations.

• “Post-mortem genetic analysis of stored tissue or DNA should be considered in deceased patients with pathologically confirmed HCM, to enable cascade genetic screening of their relatives. Class IIa, level C”.

We think that post-mortem genetic analysis should be mandatory in all patients with HCM, not only in confirmed, but also in “suspected” cases. This screening should take into consideration that HCM patients with a premature cardiovascular death may have more than one problem or reason for the disease. It is not rare to find that within a family sudden death or other adverse events have occurred in individuals with more than one disease-causing variant, and also it is possible to have a combination of two different conditions (for example HCM and long QT syndrome). The finding of a complex genotype has considerable implications for the screening and follow-up of the relatives.

The genetic study of patients with a diagnosis of “possible HCM” at the autopsy is also very important. In some cases we may find genetic variants that cause atypical forms of HCM, which may for example have a high risk of arrhythmic sudden death^7^. In our own experience (unpublished) we have seen several examples of sudden death in patients who were diagnosed in the autopsy as “idiopathic left ventricular hypertrophy” and were carriers of definitively pathogenic sarcomeric mutations and relatives of patients with HCM. We have also seen examples of patients with a diagnosis of small vessel disease and secondary fibrosis with mild hypertrophy that were finally carriers of a pathogenic troponin I mutation that caused HCM with mild hypertrophy and multiple sudden deaths in the family.

Recommendations of the ESC guideline on genetic testing in adult relatives^2^:

• “Cascade genetic screening after pre-test counseling is recommended in first-degree adult relatives of patients with a definite disease-causing mutation. Class I, level B”.
• “Clinical evaluation, employing ECG and echocardiography and long-term follow-up is recommended in first-degree relatives who have the same definitive disease-causing mutations as the probands. Class I, level C”.
• “First degree relatives who do not have the same definite disease-causing mutation as the proband should be discharged from further follow-up but advised to seek re-assessment if they develop symptoms or when new clinical relevant data emerge in the family. Class IIa, level B”.
• “When no definite genetic mutation is identified in the proband or genetic testing is not performed, clinical evaluation with ECG and echocardiography should be considered in first-degree adult relatives and repeated every 2–6 years (or 6–12 monthly if non-diagnostic abnormalities are present). Class IIa, level C”.

Usefulness and cost-effectiveness of cascade screening is clearly established. The main requirement is to have found a “definite disease-causing mutation”, which is the ideal situation. In real life many of the identified genetic variants are considered as variants of uncertain significance. Even when we report a variant as “likely pathogenic” we have to understand that this is not the same as a “definite disease-causing mutation”. Genetic counsellors often ask whether a genetic variant can be used for predictive testing, and in some cases only “predictive” tests are reimbursed.

In medicine we always have to deal with uncertainty and medical practice is based in the interpretation of probabilities. In many instances we have to objectively classify a variant as still of uncertain significance, but possibly or likely associated with the condition. In such cases the study of the co-segregation of the genetic variant with disease expression in relatives should be strongly considered, and the evaluation should always consider the inclusion of older first-degree relatives.

The identification of a candidate variant in unaffected young individuals is not particularly informative, but its presence in older ones may be a strong argument against their pathogenic role. Not performing studies of “likely” or “potentially” pathogenic variants in relatives because they “will not be predictive” is a potential mistake. The cost of such studies is very low, they provide very relevant information that may be clue for the subsequent management not only of members of the evaluated family, but also for other individuals, and with an appropriate counseling and interpretation the process should not result in any damage for the evaluated relatives. On the other side, the evaluation of the co-segregation of variants of uncertain significance that have a very low probability of being related to the condition should not be performed.

Should we make a diagnosis of the disease in a clinically unaffected relative with a “likely pathogenic” variant? The answer is no, even if the variant is clearly associated with the disease. Many pathogenic variants show incomplete penetrance: not all the carriers develop the phenotype and the disease may appear late in life.

How should we follow-up an unaffected relative with a “likely pathogenic” variant? This depends on the level of suspicion of the relation of the variant with the disease, on the severity of the expected phenotype, and on the clinical findings in the relative (completely normal vs. non-diagnostic findings that could be early manifestations of the disease).

Recommendations of the ESC guideline on genetic testing in children relatives:

• “The children of patients with a definite disease-causing mutation should be considered for genetic testing -following pre-test family counseling- when they are aged 10 or more years and this should be carried out in accordance with international guidelines for genetic testing in children. Class IIa, level C”.
• “In first-degree child relatives aged 10 or more years, in whom the genetic status is unknown, clinical assessment with ECG and echocardiography should be considered every 1–2 years between 10 and 20 years of age, and then every 2–5 years thereafter. Class IIa, level C”.
• “If requested by the parent(s) or legal representative(s) clinical assessment with ECG and echocardiography may precede or be substituted for genetic evaluation after counseling by experienced physicians and when it is agreed to be in the best interest of the child. Class IIb, level C”.
• “When there is a malignant family history in childhood or early-onset disease or when children have cardiac symptoms or are involved in particularly demanding physical activity, clinical or genetic testing of first-degree child relatives before the age of 10 years may be considered. Class IIb, level C”.

There is general agreement on the role of clinical and genetic testing in children older than 10 years. It is not frequent to develop disease-associated manifestations in children under that age. However, there are exceptional cases that should be considered. The guideline makes a class IIb recommendation on this sense: cases with malignant family history or early presentation, presence of symptoms and involvement in particularly demanding physical activity. Even though it is rare, we should not forget that children of patients with HCM might have received a second mutation. Sometimes we may suspect this possibility because this has already happened in the family, or in cases with consanguinity (that should be consider as a clear indication for early genotyping)^8^. But in small families we may fail to identify the unfortunate occurrence of a second mutation.

Malignant family history may be related either to the presence of more than one pathogenic variant in the family (complex genotypes), or to the presence of a genetic variant associated with a very severe disease expression. We have examples of variants in the literature that have caused sudden death as the first clinical manifestation in young children (for example the MYH7 Arg719Trp or Gly716Arg variants)^9^.

The identification of high-risk variants in the index case should trigger an earlier evaluation of the children even if there is no previous malignant history in their family. One controversial aspect is the comment about “particularly demanding physical activity” as a potential indication for earlier genetic testing in children. We do not think that this should be considered at the same level of indication as the presence of a severe variant in the index case or consanguinity in the family. All children perform physical activity and it is difficult to establish what “particularly demanding” means at these ages.

### Genetic diagnosis requires genotyping and interpretation

From a clinical perspective, HCM is a very heterogeneous condition, and this clinical variability is greatly dependent on the genetic heterogeneity of the disease. Sarcomeric gene mutations are the most frequent causes of the disease, and sometimes HCM is defined as a disease of the sarcomere. However, mutations in many other genes may be responsible for the development of HCM. A comprehensive HCM screening should consider genes of RASopathies (Noonan, Costello, cardiofaciocutaneous syndromes), mitochondrial proteins (mitochondrial genome or nuclear genome), transcription factors, intermediate filaments (DES, FLNC, and others), calcium regulation proteins (PLN), glycogen storage diseases (Danon disease, PRKAG2, Pompe), glycoesphingolipidosis (Fabry), amyloidosis (TTR), and many others^2,4,5,10–18^. In Table 1 we provide a list of genes currently included in our own HCM screening panel.

Until recently, a comprehensive genotyping of all these genes was not a feasible approach. Sanger techniques have a limited capacity for evaluation of candidate genes and it was essential to focus in the genes and patients with a higher pre-test probability of giving a positive result^3,4^.

In young patients with typical asymmetrical septal hypertrophy, the evaluation of the main sarcomeric genes (i.e., MYH7, MYBPC3, TNNT2, TNNI3, ACTC, MYL2, MYL3, TPM1 and TNNC) usually identified a disease causing variant in approximately 60% of the cases, while the yield of the same test in elderly patients with a septal bulge, or with concentric hypertrophy was much lower (between 10 and 30%)^19^. Some clinical “red flags” were used to focus on specific genes. Metabolic and mitochondrial conditions are more relevant in children. Concentric hypertrophy and different extra-cardiac manifestations are also associated with metabolic conditions. Pre-excitation and conduction disease put the focus on LAMP2 (Danon disease), PRKAG2, TTR (amyloidosis) or GLA (Fabry disease).

Different rare conditions have specific manifestations that give clues for a focused approach. However, in many cases the differential diagnosis is not easy and there are many atypical presentations. It is difficult to acquire clinical expertise in all these rare conditions, and mistakes based in a wrong clinical interpretation are common. As an example, Fabry disease has been in many cases over-diagnosed in individuals that were tested for the disease because of the presence of unspecific “red flags” (approximately 0.5 to 1 % of individuals in the general population are carries of GLA rare variants that are associated with moderate decreases in enzymatic activity that are not associated with disease development)^20^.

Next generation sequencing has triggered a complete change in our strategies for the identification of mutations associated with cardiomyopathies, as we can now analyse a large number of genes, or even the whole exome or genome, at a reasonable cost.

**Figure 1.**
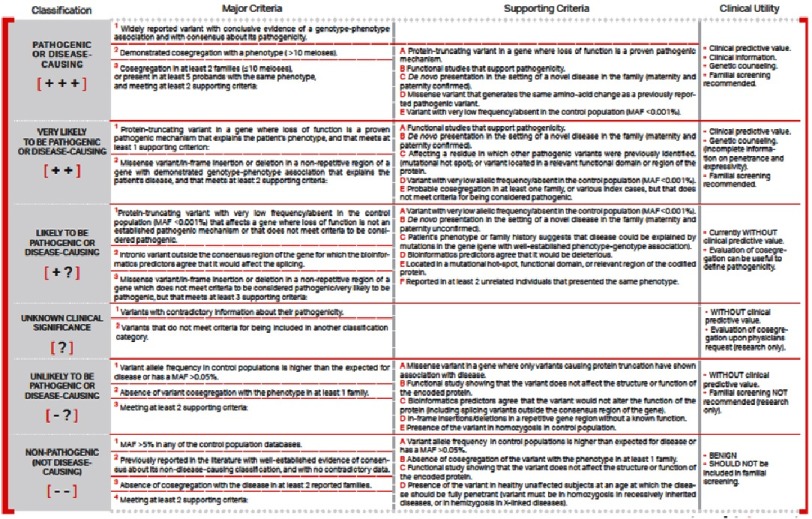
Summary of criteria for the evaluation of the pathogenicity of genetic variants. Modified from the criteria of the American Board of Medical Genetics.

As the technological capacity to identify genetic variants increases, the main limitation for the application of genetics in clinical practice is in our capacity to interpret the results. At present we study more than 200 genes per patient with our inherited cardiovascular diseases panel. These studies identify more than one thousand genetic variants in each patient. We need to classify all these variants and identify those that are pathogenic or likely pathogenic. This process requires the collaboration of molecular biologists, bioinformatics and clinicians to combine basic, epidemiologic and clinical information. We have previously described this process in detail^4,5^. In Figure 1 we summarize our criteria for the evaluation of the pathogenicity of genetic variants, which in general are in accordance with the recommendations of the American Board of Medical Genetics^21^.

**Table 1:** Genes included in our Hypertrophic Cardiomyopathy extended panel.

GENE SYMBOL	PROTEIN NAME
**ACTC1**	Actin, alpha cardiac muscle 1
**DES**	Desmin
**FLNC**	Filamin-C
**GLA**	Alpha galactosidase A
**LAMP2**	Lysosome-associated membrane glycoprotein 2
**MYBPC3**	Myosin-binding protein C, cardiac-type
**MYH7**	Myosin-7
**MYL2**	Myosin regulatory light chain 2, ventricular/cardiac muscle isoform
**MYL3**	Myosin light chain 3
**PLN**	Cardiac phospholamban
**PRKAG2**	5′-AMP-activated protein kinase subunit gamma-2
**PTPN11**	Tyrosine-protein phosphatase non-receptor type 11
**TNNC1**	Troponin C, slow skeletal and cardiac muscles
**TNNI3**	Troponin I, cardiac muscle
**TNNT2**	Troponin T, cardiac muscle
**TPM1**	Tropomyosin alpha-1 chain
**TTR**	Transthyretin
AARS2	Alanine–tRNA ligase, mitochondrial
ACAD9	Acyl-CoA dehydrogenase family member 9, mitochondrial
ACADVL	Very long-chain specific acyl-CoA dehydrogenase, mitochondrial
ACTA1	Actin, alfa 1, skeletal muscle
ACTN2	Alpha-actinin-2
AGK	Acylglycerol kinase, mitochondrial
AGL	Glycogen debranching enzyme
AGPAT2	1-acyl-sn-glycerol-3-phosphate acyltransferase beta
ANK2	Ankyrin 2
ANKRD1	Ankyrin repeat domain-containing protein 1
ATP5F1E	ATP synthase subunit epsilon, mitochondrial
ATPAF2	ATP synthase mitochondrial F1 complex assembly factor 2
BRAF	Serine/threonine-protein kinase B-raf
BSCL2	Seipin
CALR3	Calreticulin 3
CAV3	Caveolin 3
COA5	cytochrome c oxidase assembly factor 5
COA6	cytochrome c oxidase assembly factor 6 homolog
COQ2	4-hydroxybenzoate polyprenyltransferase, mitochondrial
COX15	Cytochrome c oxidase assembly protein COX15 homolog
COX6B1	Cytochrome c oxidase subunit 6B1
CRYAB	Alpha-crystallin B chain
CSRP3	Cysteine and glycine-rich protein 3
DLD	Dihydrolipoyl dehydrogenase, mitochondrial
DSP	Desmoplakin
ELAC2	Zinc phosphodiesterase ELAC protein 2
FAH	Fumarylacetoacetase
FHL1	Four and a half LIM domains protein 1
FHL2	Four and a half LIM domains 2 (FHL-2), Skeletal muscle LIM-protein 3 (SLIM-3)
FHOD3	FH1/FH2 domain-containing protein 3
FOXRED1	FAD-dependent oxidoreductase domain-containing protein 1
FXN	Frataxin, mitochondrial
GAA	Lysosomal alpha-glucosidase
GFM1	Elongation factor G, mitochondrial {ECO:0000255—HAMAP-Rule:MF_03061}
GLB1	Beta-galactosidase
GNPTAB	N-acetylglucosamine-1-phosphotransferase subunits alpha/beta
GUSB	Beta-glucuronidase
HRAS	GTPase HRas
JPH2	Junctophilin 2
KRAS	GTPase KRas
LDB3	LIM domain-binding protein 3
LIAS	Lipoyl synthase, mitochondrial
LZTR1	Leucine-zipper-like transcriptional regulator 1
MAP2K1	Dual specificity mitogen-activated protein kinase kinase 1
MAP2K2	Dual specificity mitogen-activated protein kinase kinase 2
MLYCD	Malonyl-CoA decarboxylase, mitochondrial
MRPL3	39S ribosomal protein L3, mitochondrial
MRPL44	39S ribosomal protein L44, mitochondrial
MRPS22	28S ribosomal protein S22, mitochondrial
MTO1	Protein MTO1 homolog, mitochondrial
MYH6	Myosin-6
MYOM1	Myomesin-1
MYOZ2	Myozenin-2
MYPN	Myopalladin
NEXN	Nexilin
NF1	Neurofibromin
NRAS	GTPase NRas
OBSCN	Obscurin
PDHA1	Pyruvate dehydrogenase E1 component subunit alpha, somatic form, mitochondrial
PHKA1	Phosphorylase b kinase regulatory subunit alpha, skeletal muscle isoform
PMM2	Phosphomannomutase 2
RAF1	RAF proto-oncogene serine/threonine-protein kinase
SCO2	Protein SCO2 homolog, mitochondrial
SHOC2	Leucine-rich repeat protein SHOC-2
SLC22A5	Solute carrier family 22 member 5
SLC25A3	phosphate carrier protein, mitochondrial
SLC25A4	ADP/ATP translocase 1
SOS1	Son of sevenless homolog 1
SURF1	Surfeit locus protein 1
TAZ	Tafazzin
TCAP	Telethonin
TMEM70	Transmembrane protein 70, mitochondrial
TRIM63	E3 ubiquitin-protein ligase TRIM63
TSFM	Elongation factor Ts, mitochondria
TTN	Titin
VCL	Vinculin
BAG3	BAG family molecular chaperone regulator 3
CASQ2	Calsequestrin-2
CAVIN4	Caveolae-associated protein 4
IDH2	isocitrate dehydrogenase [NADP], mitochondrial
KCNJ8	ATP-sensitive inward rectifier potassium channel 8
KLF10	Krueppel-like factor 10
LMNA	Prelamin-A/C
MYLK2	Myosin light chain kinase 2, skeletal/cardiac muscle
OBSL1	Obscurin-like protein 1
PDLIM3	PDZ and LIM domain protein 3
RYR2	Ryanodine receptor 2

The interpretation of the genetic study should not end with determining the pathogenicity of the identified variants. We need to go a step further to provide information about the clinical consequences of the genetic variant, including the age of onset, and the clinical manifestations and risks associated with its presence. Only with this information we will be able to support correct genetic counselling, risk stratification and clinical management of the disease. Knowledge management and the creation of databases that include all the available relevant clinical data on patients and relatives are essential to obtain the maximum benefit from genetic testing^4,5^.

## Genetic testing and risk stratification in HCM

Sudden death risk stratification is part of the routine management of patients with HCM and one of the most challenging tasks for the clinicians. Current recommendations focus the stratification in the evaluation of clinical markers^22^: age, family history of sudden death, previous unexplained syncope, maximal left ventricular wall thickness, left ventricular outflow tract gradient and left atrial size are considered in the ESC HCM Risk-SCD calculator (http://www.doc2do.com/hcm/webHCM.html).

Other factors, such as the presence of left ventricular systolic dysfunction, abnormal blood pressure response on exercise test or the presence and extension of gadolinium late enhancement on MRI should also be considered in an individual evaluation. There is no question about the relevant role of this clinical approach, but we should also be aware of its limitations, which are commented in the following paragraphs.

Limitations of clinical risk stratification that may be counterbalanced with the inclusion of genetic testing in the risk stratification algorithms:

a) Clinical risk stratification does not consider specific aetiologies: The presence of some specific conditions (Noonan, Fabry, Amyloidosis, etc.) is an exclusion criterion for the use of the ESC risk calculator^22^. But even in patients without those diseases the lack of consideration of the specific aetiology is a relevant limitation. The algorithms and available risk calculators are based in the assumption of that all patients with non-syndromic HCM behave in a similar way, and this is not true. For example, the relation between severity of the hypertrophy and risk is not uniform for all genes and variants.

b) The identification of clinical risk factors is subjected to chance: this is applicable to unexplained syncope, non-sustained ventricular tachycardia and family history of sudden death. Unexplained syncope in the evaluated patient and previous sudden deaths in the family are usually rare findings and may depend on the contribution of variable triggers and circumstances. Non-sustained VT is typically an infrequent finding and most patients with it have only one or two episodes of the arrhythmia in 48-hour monitoring^23^. Prolonged monitoring increases the sensitivity for the detection of the arrhythmia, but it also likely decreases the predictive value of its identification.

c) Clinical risk factors vary over time: sudden death risk calculations are focused on providing an estimate of the risk for a relative short period of time (up to 5 years). But the risk profile may change from one day to other, due to the occurrence of events in the patient (syncope, arrhythmias) or in the family (sudden deaths). Maximal left ventricular outflow tract gradient is highly variable and may be triggered or increased by factors that are not directly related with the disease prognosis (such as anemia, for example). Even maximal left ventricular wall thickness and left atrial dimensions may increase in short periods of time, especially in young individuals, and it may be difficult to anticipate the rate of progression of those parameters. Risk stratification is especially complex in patients under 16 years of age, for whom the ESC risk calculator has not been validated^22^.

d) Family history may provide insufficient or misleading information about the “genetically determined” risk: we could say that a positive family history of sudden death is a good predictor that comes too late for some members of the family. The probability of having a positive history depends of the size of the family, and small families are not very informative. Information about the incidence of sudden death in other families affected by the same pathogenic variant or variants may provide very relevant information in those cases. Family history may also be misleading when there is more than one pathogenic variant in the family: sudden death in carriers of complex genotypes (homozygous, compound heterozygous or double heterozygous carriers of pathogenic variants) may lead to an overestimation of the risk in their family members. The opposite situation, with an underestimation of the risk in affected relatives, may occur when sudden death affects individuals with “de novo” mutations. In those cases the absence of previous family history does not represent a predictor of a lower risk. In the absence of a precise genetic diagnosis these scenarios cannot be identified and the risk stratification will not be accurate.

e) Clinical risk stratification should also include an evaluation of the risk for other adverse events and especially risk for heart failure or stroke, which are not considered in the described ESC SD risk calculator.

Advantages of including genetic testing results in risk stratification algorithms:

Genetic heterogeneity underlies the clinical heterogeneity of the disease and a better understanding of the consequences of mutations in each gene, and of each mutation in a given gene is necessary to facilitate clinical risk stratification. However, previous experiences in genetically oriented risk stratification have not been very positive and many authors suggest that genetic testing does not provide relevant prognostic information.

Initial experiences tried to differentiate between “malignant” and “benign” mutations, and many studies showed inconsistency in these classifications. In our opinion, this dichotomy is too simplistic and the classifications that failed were based in too limited data. Most genetic testing reports limit the scope of the interpretation to the identification of pathogenic variants, but do not enter in a discussion about the severity of the expected clinical manifestations and prognosis associated with those variants. We have previously shown how we can get relevant prognostic information from genetic testing through the systematic compilation of data about carriers and affected relatives with a given genetic variant or with variants of similar characteristics affecting a given functional region of a protein^9^.

**Figure 2.**
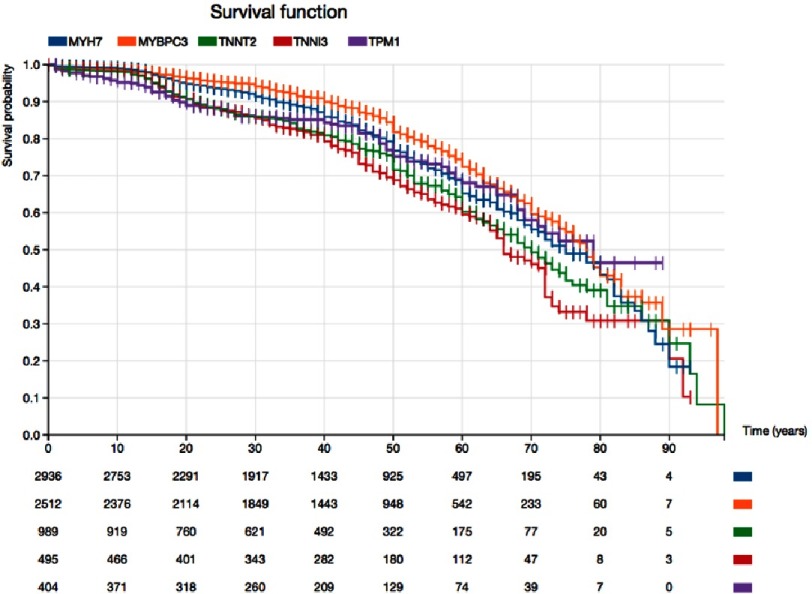
Kaplan–Meier survival curves free of sudden death, appropriate defibrillator shock, heart failure death, or cardiac transplant for patients and relatives with pathogenic or likely pathogenic variants in five of the main sarcomeric genes associated with HCM. Pathogenicity of variants was classified according to current recommendations (18 FLNC).

Since 2008, our group has dedicated considerable efforts to the systematic compilation of clinical data and outcomes of patients and relatives carrying genetic variants associated with inherited cardiovascular diseases, and in particular with HCM, collecting information of >140,000 individuals from >45,000 different families described in the international literature. More than 25,000 papers have been evaluated and included in this database, and every month we evaluate approximately 200 additional papers. Many of these publications do not provide clinical details on individual patients and this information is unfortunately lost for further analysis, but with the available data provided by publications that include clinical details we are currently able to provide relevant prognostic information for many of the identified pathogenic variants.

### Risk depends on the involved gene

In Figure 2 we provide an example of the differences between genes in survival free of cardiovascular death that we can identify through a systematic evaluation of the available published information. Here we included 2,936 individuals with pathogenic or likely pathogenic variants in MYH7, 2512 with variants in MYBPC3, 989 with variants in TNNT2, 495 with variants in TNNI3 and 404 with TPM1 variants. MYPBC3 is associated with the best prognosis, which is significantly better than that of any of the other genes. The second gene is MYH7, which is associated with a significantly better prognosis than that of TNNT2 and TNNI3. TNNI3 carriers’ survival is between 10 to 20% lower than survival for MYBPC3 carriers at different ages.

### Risk depends on the individual variants

It is evident that not all the variants in a given gene have the same clinical consequences. In any gene we may consider we will find non-pathogenic variants, variants with incomplete penetrance (sometimes with a recessive behavior) and good prognosis, and other variants that produce more severe consequences on protein’s structure and/or function. The individual clinical manifestations and prognosis depend on many factors apart from the individual mutation, and for that reason any genetic variant will have variable clinical manifestations in different individuals. For that reason, the objective is not to get from genetics a definitive conclusion about the risk of the individual, but to obtain information about the spectrum of manifestations and prognosis that could be expected. This information should be interpreted with a “probabilistic” perspective and taking into consideration all the other relevant risk markers.

**Figure 3.**
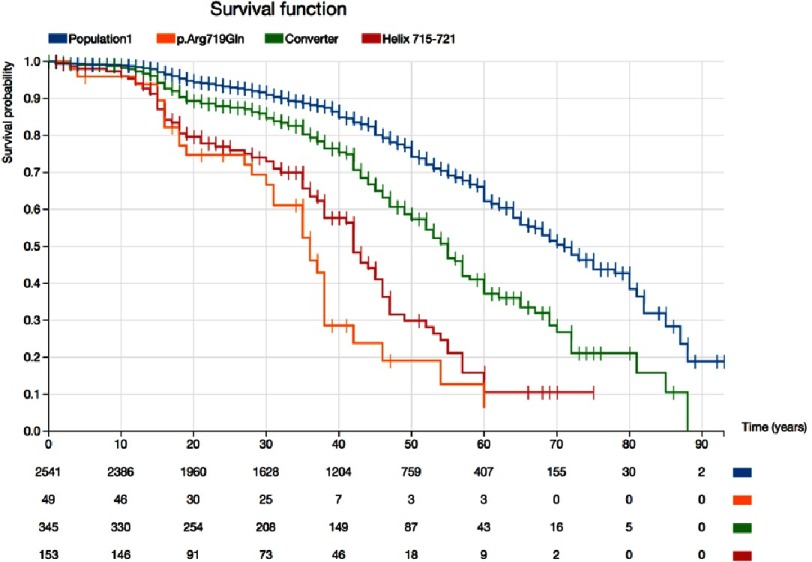
Kaplan–Meier survival curves free of sudden death, appropriate defibrillator shock, heart failure death, or cardiac transplant for patients and relatives with the Arg719Gln variant in MYH7 (orange line), compared with other variants affecting the same helix (aminoacids 715 to 721)(red line), with all pathogenic variants affecting the MYH7 converter domain (amino acids 712 to 749)(green line), and with all pathogenic missense variants in MYH7 (blue line).

The ideal situation would be to have enough information about each individual variant in order to establish the associated clinical profile, but today this is usually not possible. When the information about the consequences of a given mutation is not sufficient, we may try to compile data from mutations that could have similar consequences, and affect the same functional region^4,5,9^.

With the same methodology applied for the evaluation of prognosis for different genes, we show in Figure 3 how a detailed evaluation provides relevant information about certain genomic regions and about a particular genetic variant. The Arg719Gln variant in MYH7 affects an amino acid located in a particularly relevant alpha helix (amino acids 715 to 721), which is included in the converter domain (amino acids 712 to 749 in this example) of the beta myosin heavy chain protein^9^. Approximately 20% of the 49 described carriers and affected relatives with the Arg719Gln with available data suffered cardiovascular death (all sudden deaths) before age 20 years, and survival for this variant at age 50 was 20%. This survival was very similar to that found for 149 available individuals with 7 different pathogenic variants affecting the helix 715-721, including Gly716Arg, Asp717Val, Arg719Trp, Arg719Gln, Arg719Leu, Arg719Pro, and Arg721Lys. This survival was clearly lower than survival for the whole group of patients with pathogenic variants in the whole converter domain, which was also lower than survival for the whole group of carriers of MYH7 mutations (Figure 3).

## Therapeutic implications of genetic testing in HCM

There is a need for specific therapies in cardiomyopathies. The identification of the specific genetic defect is the first step for the understanding of the mechanisms involved in disease expression and evolution. Even though at present there are not many specific indications that come from the genetic diagnosis, some genetic diagnoses have direct therapeutic implications, like familial amyloidosis, Fabry or Pompe diseases.

As we have seen genetic testing may be very relevant for the appropriate indication of implantable defibrillators in relation to the high risk of sudden death associated with some genetic variants. Sometimes the risk is high in patients with apparently no severe clinical expression as in cases of mutations in troponin T and I mutations.

Novel therapeutic approaches may be more dependent on the identification of the specific molecular etiology of the disease. For example, the application of novel molecules that modify sarcomere contractility^24^, or calcium sensitivity^25^ could have different effects depending on the main molecular abnormalities that cause the phenotype. Genetic diagnosis is also the base for the development of specific gene-therapies that are currently under development^26^.

## Genetic testing for a better disease understanding

Even though this is usually not considered an acceptable indication for genetic testing in the clinical setting, we consider that it is essential to perform this type of testing for the patients’ benefit. Hypertrophic cardiomyopathy was first described in the fifties and we still do not know how to correctly identify patients at high risk, mainly because we have not understood the reasons behind heterogeneity, variable presentation and evolution of this disease. Only by understanding aetiology we will be able to develop novel and more specific therapies. This will not only affect key decisions such as whether to implant a defibrillator but will also provide the necessary tools to potentially modify or even abolish phenotypic expression of the disease.

## Genetic testing and genetic counseling in HCM

Genetic counselling could be defined as the process by which patients or relatives are advised of the consequences and nature of a potentially inherited disorder, the probability of developing or transmitting it, and the options open to them in management and family planning. This means that genetic counselling is much more than just to inform about the potential heritability or transmission of the disease. Successful identification of the specific genetic origin of the disease and good experience in identifying potential clinical consequences of this genetic predisposing factor are essential to provide adequate genetic counselling.

## Conclusions

Genetic testing should be routinely considered in the diagnostic and prognostic evaluation of patients with HCM and is an essential tool for the evaluation and early diagnosis of relatives at risk. The interpretation of genetic testing results is a complex process that requires the collaboration of multidisciplinary specialized teams supported by knowledge management systems that help to integrate and analyze in an efficient and personalized way all the available relevant information.

## Conflicts of interest

The author is the CEO of Health in Code. Results from their own HCM screening panel are described herein.

## References

[ref-1] Elliott P, Andersson B, Arbustini E, Bilinska Z, Cecchi F, Charron P (2007). Classification of the cardiomyopathies: a position statement from the european society of cardiology working group on myocardial and pericardial diseases. Eur Heart J.

[ref-2] Elliott PM, Anastasakis A, Borger MA, Borggrefe M, Cecchi F, Charron P (2014). 2014 ESC Guidelines on diagnosis and management of hypertrophic cardiomyopathy: The Task Force for the Diagnosis and Management of Hypertrophic Cardiomyopathy of the European Society of Cardiology (ESC). Eur Heart J.

[ref-3] Monserrat L, Mazzanti A, Ortiz-Genga M, Barriales-Villa R, Garcia D, Gimeno-Blanes JR (2011). The interpretation of genetic tests in inherited cardiovascular diseases. Cardiogenetics.

[ref-4] Monserrat L, Ortiz-Genga M, Lesende I, Garcia-Giustiniani D, Barriales-Villa R, de Una-Iglesias D (2015). Genetics of cardiomyopathies: novel perspectives with next generation sequencing. Curr Pharm Des.

[ref-5] Charron P, Arad M, Arbustini E, Basso C, Bilinska Z, Elliott P (2010). Genetic counselling and testing in cardiomyopathies: a position statement of the European Society of Cardiology Working Group on Myocardial and Pericardial Diseases. Eur Heart J.

[ref-6] Ingles J, McGaughran J, Scuffham PA, Atherton J, Semsarian C (2012). A cost-effectiveness model of genetic testing for the evaluation of families with hypertrophic cardiomyopathy. Heart.

[ref-7] Pasquale F, Syrris P, Kaski JP, Mogensen J, McKenna WJ, Elliott P (2012). Long-term outcomes in hypertrophic cardiomyopathy caused by mutations in the cardiac troponin T gene. Circ Cardiovasc Genet.

[ref-8] Zahka K, Kalidas K, Simpson MA, Cross H, Keller BB, Galambos C, Gurtz K, Patton MA, Crosby AH (2008). Homozygous mutation of MYBPC3 associated with severe infantile hypertrophic cardiomyopathy at high frequency among the Amish. Heart.

[ref-9] García-Giustiniani D, Arad M, Ortíz-Genga M, Barriales-Villa R, Fernández X, Rodríguez-García I (2015). Phenotype and prognostic correlations of the converter region mutations affecting the *β* myosin heavy chain. Heart.

[ref-10] Kindel SJ, Miller EM, Gupta R, Cripe LH, Hinton RB, Spicer RL (2012). Pediatric cardiomyopathy: importance of genetic and metabolic evaluation. J Card Fail.

[ref-11] Gollob MH, Green MS, Tang ASL, Roberts R (2002). PRKAG2 cardiac syndrome: familial ventricular preexcitation, conduction system disease, and cardiac hypertrophy. Curr Opin Cardiol.

[ref-12] Arad M, Maron BJ, Gorham JM, Johnson WH, Saul JP, Perez-Atayde AR (2005). Glycogen storage diseases presenting as hypertrophic cardiomyopathy. N Engl J Med.

[ref-13] Weidemann F, Linhart A, Monserrat L, Strotmann J (2010). Cardiac challenges in patients with Fabry disease. Int J Cardiol.

[ref-14] Monserrat L, Gimeno-Blanes JR, Marín F, Hermida-Prieto M, García-Honrubia A, Pérez I (2007). Prevalence of Fabry Disease in a Cohort of 508 Unrelated Patients With Hypertrophic Cardiomyopathy. J Am Coll Cardiol.

[ref-15] Rapezzi C, Quarta CC, Riva L, Longhi S, Gallelli I, Lorenzini M (2010). Transthyretin-related amyloidoses and the heart: a clinical overview. Nat Rev Cardiol.

[ref-16] Tartaglia M, Gelb BD, Zenker M (2011). Noonan syndrome and clinically related disorders. Best Pract Res Clin Endocrinol Metab.

[ref-17] Meyers DE, Basha HI, Koenig MK (2013). Mitochondrial cardiomyopathy: pathophysiology, diagnosis, and management. Tex Heart Inst J.

[ref-18] Rapezzi C, Arbustini E, Caforio ALP, Charron P, Gimeno-Blanes J, Heliö T (2013). Diagnostic work-up in cardiomyopathies: Bridging the gap between clinical phenotypes and final diagnosis. A position statement from the ESC Working Group on Myocardial and Pericardial Diseases. Eur Heart J.

[ref-19] Binder J, Ommen SR, Gersh BJ, Van Driest SL, Tajik AJ, Nishimura RA (2006). Echocardiography-guided genetic testing in hypertrophic cardiomyopathy: septal morphological features predict the presence of myofilament mutations. Mayo Clin Proc.

[ref-20] Ferreira S, Ortiz A, Germain DP, Viana-Baptista M, Caldeira-Gomes A, Camprecios M (2015). The alpha-galactosidase A p.Arg118Cys variant does not cause a Fabry disease phenotype: data from individual patients and family studies. Mol Genet Metab.

[ref-21] Richards S, Aziz N, Bale S, Bick D, Das S, Gastier-Foster J (2015). Standards and guidelines for the interpretation of sequence variants: a joint consensus recommendation of the American College of Medical Genetics and Genomics and the Association for Molecular Pathology. Genet Med.

[ref-22] O’Mahony C, Jichi F, Pavlou M, Monserrat L, Anastasakis A, Rapezzi C (2014). A novel clinical risk prediction model for sudden cardiac death in hypertrophic cardiomyopathy (HCM risk-SCD). Eur Heart J.

[ref-23] Monserrat L, Elliott PM, Gimeno JR, Sharma S, Penas-Lado M, McKenna WJ (2003). Non-sustained ventricular tachycardia in hypertrophic cardiomyopathy: an independent marker of sudden death risk in young patients. J Am Coll Cardiol.

[ref-24] Green EM, Wakimoto H, Anderson RL, Evanchik MJ, Gorham JM, Harrison BC (2016). A small-molecule inhibitor of sarcomere contractility suppresses hypertrophic cardiomyopathy in mice. Science.

[ref-25] Thompson BR, Martindale J, Metzger JM (2016). Sarcomere neutralization in inherited cardiomyopathy: small- molecule proof-of-concept to correct hyper-Ca2+-sensitive myofilaments. Am J Physiol Heart Circ Physiol.

[ref-26] Prondzynski M, Krämer E, Laufer SD, Shibamiya A, Pless O, Flenner F (2017). Evaluation of MYBPC3 trans-Splicing and Gene Replacement as Therapeutic Options in Human iPSC-Derived Cardiomyocytes. Mol Ther Nucleic Acids.

